# Supplementary material for: Kaempferia chonburiensis (Zingiberaceae), a new species from Thailand based on morphological and molecular evidence
Source: PeerJ. 2025 Feb 24;13:e18948. doi: 10.7717/peerj.18948 (PMC11867034; doi:10.7717/peerj.18948)
Supplement: Supplemental Information 1 — Newly generated sequences are marked in bold; –, missing data. [file peerj-13-18948-s001.docx]

**Table S1 Voucher information, location, and GenBank accession numbers for samples used in this study** **(newly generated sequences are marked in bold; –, missing data).**

| **Taxon** | **Location** | **Voucher/Source** | **ITS** | ***mat*K** | **Remark** |
| --- | --- | --- | --- | --- | --- |
| *Kaempferia angustifolia* Roscoe | Udon Thani, NE | *PW* *030721*–*1* (BKF) | **LC832953** | **LC832970** |  |
| *Kaempferia chonburiensis* sp. nov*.* 1 | Chon Buri, SE | *PW* *240621*–*1* (BKF) | **LC832954** | **LC832971** |  |
| *Kaempferia chonburiensis* sp. nov*.* 2 | Chon Buri, SE | *PW* *240621*–*2* (BKF) | **LC832955** | **LC832972** |  |
| *Kaempferia chonburiensis* sp. nov*.* 3 | Chon Buri, SE | *PW* *240621*–*3* (BKF) | **LC832956** | **LC832973** |  |
| *Kaempferia elegans* Wall. | Kanchanaburi, SW | *PW* *210722*–*1* (BKF) | **LC832957** | **LC832974** |  |
| *Kaempferia fissa* Gagnep. | Ubon Ratchathani, E | *PW* *230723*–*1* (BKF) | **LC832958** | **LC832975** |  |
| *Kaempferia galanga* L. | Uttaradit, N | *PW* *060722*–*1* (BKF) | **LC832959** | **LC832976** | Cultivated |
| *Kaempferia koratensis* Picheans. | Nakhon Ratchasima, E | *PW* *240721*–*1* (BKF) | **LC832960** | **LC832977** |  |
| *Kaempferia larsenii* Sirirugsa | Ubon Ratchathani, E | *PW* *170622*–*1* (BKF) | **LC832961** | **LC832978** |  |
| *Kaempferia marginata* Carey ex Roscoe | Si Sa Ket, E | *PW* *250723*–*1* (BKF) | **LC832962** | **LC832979** |  |
| *Kaempferia minuta* Jenjitt. & K. Larsen | Ubon Ratchathani, E | *PW* *090622*–*1* (BKF) | **LC832963** | **LC832980** |  |
| *Kaempferia parviflora* Wall. ex Baker | Chon Buri, SE | *PW* *210723*–*1* (BKF) | **LC832964** | **LC832981** | Cultivated |
| *Kaempferia pulchra* Ridl. | Phangnga, PEN | *PW* *070722*–*1* (BKF) | **LC832965** | **LC832982** |  |
| *Kaempferia roscoeana* Wall. | Kanchanaburi, SW | *PW* *220721*–*1* (BKF) | **LC832966** | **LC832983** |  |
| *Kaempferia rotunda* L. | Chon Buri, SE | *PW* *030422*–*1* (BKF) | **LC832967** | **LC832984** |  |
| *Kaempferia sisaketensis* Picheans. & Koonterm | Si Sa Ket, E | *PW 010723-1* (BKF) | **LC832968** | **LC832985** |  |
| *Kaempferia udonensis* Picheans. & Phokham | Udon Thani, NE | *PW* *280322*–*1* (BKF) | **LC832969** | **LC832986** |  |
| *Zingiber wrayi* | **–** | Kress et al. (2002) | AF478802 | AF478905 |  |

**Floristic regions of Thailand.** N = Northern; NE = Northeastern; E = Eastern; SW = Southwestern; C = Central; SE = Southeastern; PEN = Peninsular.

**Kress WJ, Prince LM, Williams KJ. 2002.** The phylogeny and a new classification of the gingers (Zingiberaceae): evidence from molecular data. *American journal of botany.* **89(10):**1682–1696 DOI 10.3732/ajb.89.10.1682.
