# Supplementary material for: Kaempferia chonburiensis (Zingiberaceae), a new species from Thailand based on morphological and molecular evidence
Source: PeerJ. 2025 Feb 24;13:e18948. doi: 10.7717/peerj.18948 (PMC11867034; doi:10.7717/peerj.18948)
Supplement: Supplemental Information 2 [file peerj-13-18948-s002.docx]

**Table S2 The coding of morphological characteristics used for character state analysis.**

| **Species** | **Floristic regions** | **Inflorescence** | **Rhizome** | **Ligule** | **Leaves** | **Peduncle** | **Staminodes and labellum** |
| --- | --- | --- | --- | --- | --- | --- | --- |
| *Kaempferia angustifolia* Roscoe | Udon Thani, NE | with leaves | non monoliform | absent-5 mm | non horizontal | sessile-5 mm | not same plane |
| *Kaempferia chonburiensis* sp. nov*.* | Chon Buri, SE | with leaves | monoliform | >5 mm | horizontal | >5 mm | same plane |
| *Kaempferia elegans* Wall. | Kanchanaburi, SW | with leaves | monoliform | absent-5 mm | non horizontal | >5 mm | same plane |
| *Kaempferia fissa* Gagnep. | Ubon Ratchathani, E | with leaves | non monoliform | absent-5 mm | non horizontal | sessile-5 mm | same plane |
| *Kaempferia galanga* L. | Uttaradit, N | with leaves | monoliform | absent-5 mm | horizontal | sessile-5 mm | same plane |
| *Kaempferia koratensis* Picheans. | Nakhon Ratchasima, E | with leaves | monoliform | absent-5 mm | horizontal | sessile-5 mm | same plane |
| *Kaempferia larsenii* Sirirugsa | Ubon Ratchathani, E | with leaves | monoliform | absent-5 mm | non horizontal | sessile-5 mm | same plane |
| *Kaempferia marginata* Carey ex Roscoe | Si Sa Ket, E | with leaves | monoliform | absent-5 mm | horizontal | sessile-5 mm | not same plane |
| *Kaempferia minuta* Jenjitt. & K. Larsen | Ubon Ratchathani, E | with leaves | monoliform | absent-5 mm | horizontal | sessile-5 mm | same plane |
| *Kaempferia parviflora* Wall. ex Baker | Chon Buri, SE | with leaves | monoliform | absent-5 mm | non horizontal | >5 mm | not same plane |
| *Kaempferia pulchra* Ridl. | Phangnga, PEN | with leaves | monoliform | absent-5 mm | horizontal | >5 mm | same plane |
| *Kaempferia roscoeana* Wall. | Kanchanaburi, SW | with leaves | monoliform | absent-5 mm | horizontal | sessile-5 mm | same plane |
| *Kaempferia rotunda* L. | Chon Buri, SE | before leaves | non monoliform | absent-5 mm | non horizontal | sessile-5 mm | not same plane |
| *Kaempferia sisaketensis* Picheans. & Koonterm | Si Sa Ket, E | with leaves | non monoliform | absent-5 mm | non horizontal | sessile-5 mm | staminodes absent |
| *Kaempferia udonensis* Picheans. & Phokham | Udon Thani, NE | before leaves | non monoliform | >5 mm | horizontal | sessile-5 mm | same plane |
